# Microarray-Based Capture of Novel Expressed Cell Type–Specific Transfrags (CoNECT) to Annotate Tissue-Specific Transcription in *Drosophila melanogaster*

**DOI:** 10.1534/g3.112.003194

**Published:** 2012-08-01

**Authors:** X. Hong, H. Doddapaneni, J. M. Comeron, M. J. Rodesch, H. A. Halvensleben, C. Y. Nien, F. Bolei, R. Metpally, T. A. Richmond, T. J. Albert, J. R. Manak

**Affiliations:** * Department of Biology, University of Iowa, Iowa City, Iowa 52242; †† Department of Pediatrics, University of Iowa, Iowa City, Iowa 52242; † Carver Center for Genomics, University of Iowa, Iowa City, Iowa 52242; ‡ Roche NimbleGen, Madison, Wisconsin 53719; § Department of Biology, Center for Developmental Genetics, New York University, New York, New York 10003, and; ** Translational Genomics Research Institute, Phoenix, Arizona 85004

**Keywords:** transcriptome, array capture, enrichment, transcript isoforms

## Abstract

Faithful annotation of tissue-specific transcript isoforms is important not only to understand how genes are organized and regulated but also to identify potential novel, unannotated exons of genes, which may be additional targets of mutation in disease states or while performing mutagenic screens. We have developed a microarray enrichment methodology followed by long-read, next-generation sequencing for identification of unannotated transcript isoforms expressed in two *Drosophila* tissues, the ovary and the testis. Even with limited sequencing, these studies have identified a large number of novel transcription units, including 5′ exons and extensions, 3′ exons and extensions, internal exons and exon extensions, gene fusions, and both germline-specific splicing events and promoters. Additionally, comparing our capture dataset with tiling array and traditional RNA-seq analysis, we demonstrate that our enrichment strategy is able to capture low-abundance transcripts that cannot readily be identified by the other strategies. Finally, we show that our methodology can help identify transcriptional signatures of minority cell types within the ovary that would otherwise be difficult to reveal without the CoNECT enrichment strategy. These studies introduce an efficient methodology for cataloging tissue-specific transcriptomes in which specific classes of genes or transcripts can be targeted for capture and sequence, thus reducing the significant sequencing depth normally required for accurate annotation.

Over the last few years, it has become evident that a significant percentage of genes in higher eukaryotic genomes encode a multitude of tissue-specific transcript isoforms expressed in temporospatially distinct patterns (Gan *et al.* 2010; Graveley *et al.* 2011; Manak *et al.* 2006). To comprehensively annotate a genome and understand the regulation of such complex gene structures, it is important to identify all transcript isoforms. Recently, several studies have attempted to profile the transcriptome of specific tissues or developmental stages in a variety of higher eukaryotes using several genomic methodologies, including tiled genomic microarrays, RNA-seq, and Sanger sequencing of cDNA libraries (Cherbas *et al.* 2011; Costa *et al.* 2010; Gan *et al.* 2010; Graveley *et al.* 2011; Malone and Oliver 2011; Manak *et al.* 2006; Wan *et al.* 2006). RNA-seq is the most high-throughput and efficient methodology of the three approaches, and it ultimately relies upon cDNA sequencing using next-generation sequencing platforms, such as Illumina, SOLiD, or 454 (Malone and Oliver 2011; Wang *et al.* 2009). The first two sequencing platforms generate large numbers of short-read sequences, whereas the 454 platform generates fewer but significantly longer read sequences (Metzker 2010). However, the standard RNA-seq procedures that utilize all of these platforms have numerous shortcomings. First, to prepare cDNA libraries for next-generation sequencing, steps are needed to remove rRNA (Wilhelm and Landry 2009), as without such removal these rRNAs would contribute to a large percentage of the sequencing reads, thus reducing the ability to more deeply sequence the mRNA pool. Additionally, other undesirable RNAs, which might include highly expressed housekeeping genes, can make up a significant percentage of reads from an mRNA sample, once again hindering the ability to probe more deeply into the transcriptome. Second, we and others have found that for many standard RNA-seq experiments, even when performed using published “standards” of read number and depth coverage, such experiments often fail to fully “cover” low-expressed or even moderately expressed genes, leaving “holes” in the gene structures (Graveley *et al.* 2011; Malone and Oliver 2011). Thus, to interrogate low-level transcripts, a larger number of sequencing reads need to be generated, which can make the methodology cost prohibitive. Third, for transcript isoforms expressed in a small subset of cells in the tissue or organism of interest, it may be difficult to characterize the transcripts specific to those cells (Graveley *et al.* 2011; Malone and Oliver 2011). For example, if only a few progenitor heart cells begin expressing heart-specific genes in the context of a much larger group of cells, such transcripts might go undetected; this would result in a failure to identify the true initiation of heart-specific gene transcription. Fourth, for transcriptionally complex genes encoding many different cell-specific isoforms, it can be difficult to identify all the relevant transcript isoforms.

To overcome such limitations, we developed an array-based sequence capture strategy that we call CoNECT (capture of novel expressed cell type–specific transfrags) in which we enriched for tissue-specific cDNA fragments [transcribed fragments, or transfrags, (Manak *et al.* 2006)] using exome microarrays followed by 454 sequencing. Given the rich diversity of transcript isoforms hypothesized to be expressed in the germline, we chose to focus on *Drosophila melanogaster* testes and ovaries (Kai *et al.* 2005). We used a 454 sequencing platform specifically because it generates long read lengths; indeed, in our experience, novel 5′ ends (which make up a substantial percentage of transcript diversity) can extend several hundred bases from an annotated exon, sometimes containing multiple exons. We thus wanted the capability of generating the longest length of sequence possible to faithfully annotate these exons and their appropriate splice junctions. Exome microarrays and related methodologies have recently been used to enrich exonic regions of genomic DNA for identification of disease-causing mutations in humans (Bamshad *et al.* 2011; Pierson *et al.* 2011) and mice (Fairfield *et al.* 2011; Hilton *et al.* 2011). We wanted to explore whether this technology could be adapted to cDNA enrichment and sequencing to identify novel transcript isoforms that had previously gone undetected in these tissues. Recently, Mercer and colleagues (Mercer *et al.* 2012) used a similar strategy to capture unannotated transcripts in specific regions of the genome. However, their study focused on tiling array capture and characterization mostly of novel transcripts, whereas the study described here focuses on characterizing the novel isoforms in the protein-coding transcriptome across the entire genome through utilization of exome arrays. Validation of such a method would allow exome enrichment platforms already in the marketplace to be adapted for this purpose, as well as pave the way for the use of more specific exon-based arrays that seek to interrogate a limited number of genes. We report here on the proof of principle for this methodology and demonstrate that novel transcript isoforms can be effectively identified while minimizing cost. The study also demonstrates that CoNECT can identify genes expressed in small subsets of cells within a tissue.

## Materials and Methods

### Fly strain, tissue dissection, RNA extraction, and cDNA synthesis

The fly strain we used in this study is *Drosophila melanogaster* [*yellow* (*y*); *cinnabar* (*cn*); *brown* (*bw*); *speck* (*sp*)] used in the BDGP sequencing project (Celniker *et al.* 2002). Fly embryos were collected using small grape plates (0–4 hr, 25°) at day 0 and raised at 25°. Eclosing virgin females and males were collected into different bottles containing standard media from day 9 to day 10. Ovaries and testes were dissected in 1× PBS solution under a dissecting microscope from day 11 to day 12 (1–2 days after eclosion). Dissected ovaries and testes were quickly frozen in Trizol reagent at −80°. Total RNA was isolated from ovaries and testes following the Trizol RNA isolation protocol (Invitrogen) and purified on RNeasy columns (Qiagen). mRNAs were isolated using the Poly(A) purist kit (Ambion) and then transcribed into double-stranded cDNA using the Superscript Double-Stranded cDNA synthesis kit following the manufacturer's instructions (Invitrogen). Six µg of ovary and 5.7 µg of testis cDNA were sent to Nimblegen for capture.

### CoNECT array design

The collection of all known and predicted exons in *D*. *melanogaster* r5.19 was downloaded from FlyBase (ftp://ftp.flybase.net/genomes/Drosophila_melanogaster/dmel_r5.19_FB2009_06/fasta/ dmel-all-exon-r5.19.fasta and /dmel-all-predicted-r5.19.fasta). Target coordinates from these sets were consolidated to combine overlapping target regions, resulting in 83,616 target regions totaling 34,963,568 bp. Unique probes targeting these coordinates were selected against *D. melanogaster* r5.7, utilizing a rebalancing algorithm to improve uniformity of coverage across targets as previously described (Bainbridge *et al.* 2010), resulting in 2,160,993 probes targeting 81,872 regions covering 34,418,101 bp.

### CoNECT procedure

*Drosophila* ovary and testis cDNA samples were checked for quality and degradation using an Agilent 2100 Bioanalyzer with a DNA 7500 chip. Five hundred nanograms of each cDNA sample was used for library preparation following 454 LifeSciences *Rapid Library Preparation Method Manual* (http://dna.uga.edu/docs/GS-FLX-Titanium_RapidLibrary%20%28RL%29%20Preparation_Method_Manual%20%28Roche%29.pdf). After preparation via the 454 protocol, samples were eluted in PCR-grade water and subjected to precapture ligation-mediated PCR (LM-PCR) using the Roche FastStart High Fidelity PCR system (cat no. 04738292001). Each reaction contained 10 µl FastStart buffer, 2 µl DMSO, 2 µl PCR nucleotide mix, 10 µl each of oligos LM-PCR 454 Ti-A: 5′–CCATCTCATCCCTGCGTGTC–3′ and LM-PCR 454 Ti-B: 5′–CCTATCCCCTGTGTGCCTTG–3′ at 40 µM concentration, 15 µl PCR-grade water, 50 µl cDNA sample, and 1 µl FastStart High-Fidelity Enzyme Blend. Reactions were subjected to 95° for 10 min, followed by 30 sec at 95°, 30 sec at 64°, 3 min at 72°, with repetition of steps 2–4 for 11 cycles, followed by 7 min at 72° and then held at 4°. Amplicons were then purified using a Qiagen Qiaquick column with elution in 50 µl of water. Sample hybridization was performed on the Roche NimbleGen custom 2.1M *Dropsophila* exome array described above. Hybridization cocktail containing 2 µg of precapture library with 300 µg of Cot-1 DNA was dried on high heat (60°). Once dried, 1 µl of each hybridization-enhancing oligo was added (R-A Hyb Enhancing: 5′–CCATCTCATCCCTGCGTGTCTCCGACGACT–3′ and R-B Hyb Enhancing: 5′–CCTATCCCCTGTGTGCCTTGCCTCCCACGACT–3′ at 1000 µM concentration), in addition to 11.2 µl of PCR-grade water, 18.5 µl of Nimblegen 2× hybridization buffer, 7.3 µl of NimbleGen Hybridization Component A. Sample tubes were mixed and heated at 95° for 10 min. Hybridization cocktail was loaded onto the array using an HX1 mixer and NimbleGen Hybridization Station according to manufacturer's instructions and left to mix at 42° for 68 hr. Slide washing and sample elution were performed as previously described (Fu *et al.* 2010). Eluted samples were again subjected to LM-PCR as above with samples divided into four reactions to minimize potential amplification bias. Postcapture LM-PCR consisted of 20 cycles, after which samples were purified using Qiagen Qiaquick columns. Captured samples were then sequenced using the Roche 454 Genome Sequencer FLX system.

### Identification of novel transcript isoforms

Newbler (v2.3) was used to assemble the 454 reads into isotigs or singletons. To identify new isoforms, we used the RepeatMasker program version 3.2.9 and the NCBI/RMBLAST that is part of the program as search engine. The RepeatMasker program ordinarily screens DNA sequences for interspersed repeats and low complexity DNA sequences against a provided database of known repeat sequences; in this case, instead of using a repeat sequence database, fasta sequences of known *D*. *melanogaster* genes or the genome were used as a database for screening. This leads to the masking of the complementary regions of the known genes in the sequenced dataset, and the regions left unmasked (either 5′, internal, or 3′ to the masked sequence) were treated as potential novel sequences for further evaluation. Specifically, two rounds of screening using the RepeatMasker program were carried out to identify novel exons and extensions using the default program settings. In the first round, isotigs and singletons were screened against the *D*. *melanogaster* all-genes database (dmel-all-gene-r5.33.fasta) to determine sequences that are fully masked, suggesting that they are fully known transcripts, as well as partially masked sequences that may include a novel extension/insertion. A sequence was considered as a potential new isoform if the region left unmasked was at one end of the sequencing read and was greater than 10 bp long, or it was inside the masked sequence. In the second round, these partially masked sequences were screened against the *D*. *melanogaster* genome (dmel-all-chromosome-r5.33.fasta) to identify the genomic location of these sequences. Depending on the matching positions of the sequences and their unmasked region, seven different categories of novel exons or extensions can be determined, including 5′ novel exons, 5′ exon extensions, 3′ novel exons, 3′ exon extensions, novel internal exons, internal exon extensions, and gene fusions. Raw sequence data have been submitted to the sequence read archive (SRA), accession number SRA050707.2. Ovary and testis isotigs over 200 bp have been submitted to the Transcriptome Shotgun Assembly Sequence Database in GenBank The bioproject number is PRJNA89451, and the accession numbers are JV208106–JV230865. Singleton sequences, as well as isotigs less than or equal to 200 bp, are included as supporting information for the ovary (File S1 and File S2) and testis (File S3 and File S4) datasets.

### Tiling array gene expression analysis

The custom-designed *Drosophila melanogaster* tiled genomic microarray has been previously described (Nien *et al.* 2011). This array set, which comprises two HD2 (2.1 million feature) microarrays, utilizes 50-mer oligonucleotide probes with up to 100 close matches per sequence tolerated and a median probe spacing of 33 bp. After hybridization, processing, and scanning of three technical replicate array sets, intensity readings of probes were corrected according to their GC content using a set of random probes on the arrays, and then normalized by quantile normalization (Nien *et al.* 2011). After normalization, a median filter was applied. The expression level of each exon was calculated by taking the median of all the probes covering the exon, and the expression level of each RNA isoform was calculated by averaging the expression levels of all the exons of the isoform without weighting. The background threshold was set as 2% FDR (calculated by “fdrtool” package). Genes were considered as expressed (present) if more than 70% of the probe signals were higher than the threshold; otherwise, they were considered as not expressed (absent) (Nien *et al.* 2011) (Table S10). Genes that were present in all three technical replicates were considered as expressed and further used to compare with genes identified by CoNECT.

To make the GFF files visualized in the SignalMap genome browser, tiling array data were quantile normalized and median filtering was applied on probes from the three replicates employing a sliding window of three probes [assigning the median value to the center probe from the three replicates (3×3)]. Background subtraction was performed by subtracting the top 2% of random probe (negative control) intensity values from individual *D. melanogaster* probe intensity values. GFF files were generated after the probes with negative intensity values (after background subtraction) were rounded to zero (see GFF files in File S5, File S6, and File S7).

### Selective capture array enrichment analysis

To obtain CoNECT expression data directly comparable to tiling array gene expression and traditional RNA-seq (Gan *et al.* 2010) datasets, we determined the FPKM values based on 454 sequencing reads generated by CoNECT. We first mapped 454 reads to the whole list of *D. melanogaster* transcripts (dmel-all-transcript-r5.45.fasta) using BWA-SW (Li and Durbin 2010) and then obtained FPKM values per transcript based on the best match per read and using samtools idxstats (Li *et al.* 2009a). Finally, we obtained FPKM values per gene by collapsing into one all alternative transcripts when present.

To determine whether CoNECT effectively enriches for targeted transcripts at the expense of transcripts that were not represented in the capture array design, we investigated the number of reads corresponding to the duplicated gene *ada1* (*ada1-1*, FBgn0051865; *ada1-2*, FBgn0051866), which showed strong expression on the tiling array but was not interrogated by any probes on the capture array, and the 10 most closely expressed genes determined via the tiling array analysis (Table S12). The number of 454 reads that mapped to *ada1* and the 10 most closely expressed genes are listed in Table S12 (along with their FPKM values). Importantly, any reads with sequences identical to two locations in the genome were not removed and one (the best match) was chosen; thus, no reads mapping to *ada1* would have been discarded from both duplicated copies. To test for a significant reduction of *ada1* reads, we took into account the differences in each gene transcript size among the 11 genes.

### David functional annotation clustering

Nine-hundred seventy-four capture-specific genes were uploaded onto David Bioinformatics Resources 6.7, and 135 clusters were generated using the default option setting and stringency (medium). We removed clusters and terms under each annotation cluster that had *P*-values larger than 0.05; Table S13 lists 42 enriched clusters with significant *P*-values. The enrichment score of each annotation cluster is the geometric mean (in -log scale) of the member's *P*-value and is used to rank its biological significance (Huang da *et al.* 2009). Also, 32 neuro-related GO terms covering 63 genes were identified by looking through all 135 clusters, regardless of the *P*-value scores. Among these 32 terms, 4 terms under cluster 10, including neurotransmitter receptor activity, neurotransmitter binding, neuropeptide binding, and neuropeptide receptor activity, had significant *P*-values, which are indicated in the parentheses after each term (Table S14).

## Results

### Capture of expressed transcripts

**Figure 1:**
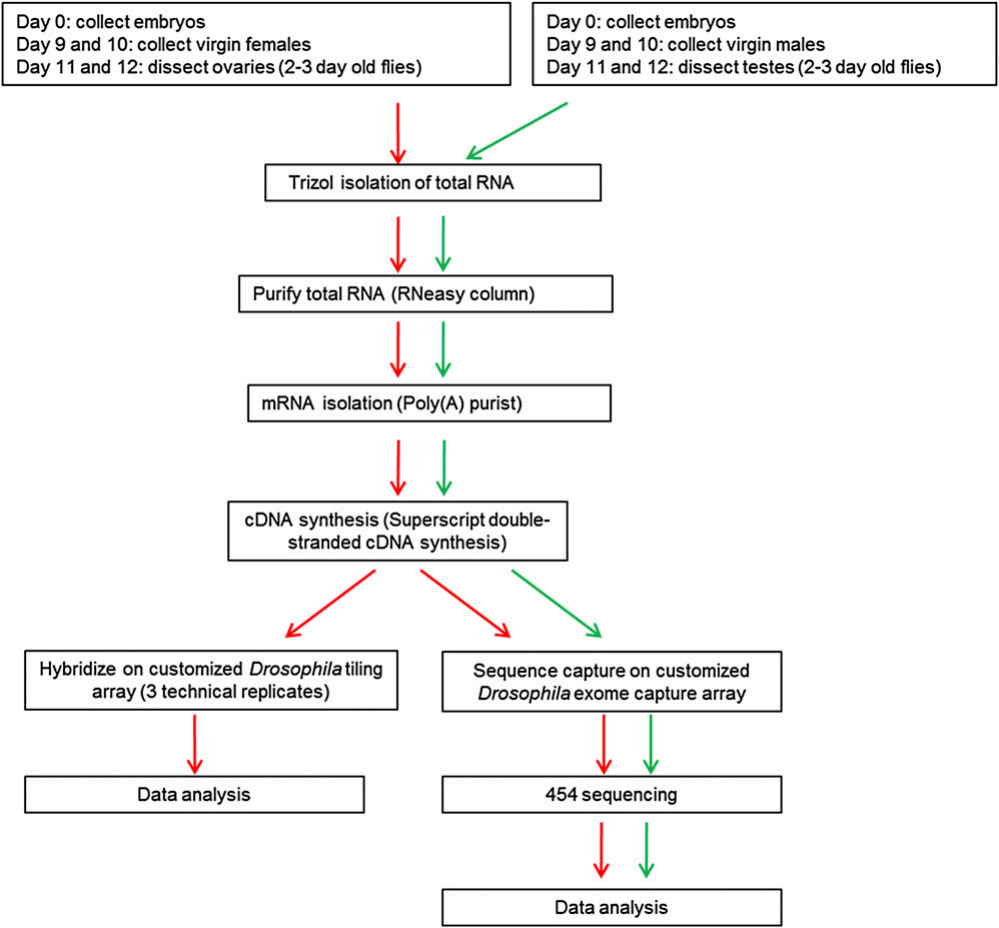
Pipeline underlying the experimental design. Ovary sample (indicated by red arrows) was processed for both tiling array (bottom left) and sequence capture array (bottom right). Testis sample (indicated by green arrows) was used only for sequence capture analysis.

A 2.1 million feature Roche NimbleGen microarray targeting 81,872 annotated exons from *Drosophila* release r5.7 was designed using synthesis cycles that produced oligonucleotides of up to 106 bases, with an average size of 76 bases. Total RNA was isolated from approximately 16,000 testes and 6000 ovaries and mRNA was purified, followed by double-stranded cDNA synthesis (Figure 1). After nebulization of the cDNA followed by library preparation and LM-PCR, the cDNA fragments were hybridized to the array. We used nebulization parameters that produced cDNA fragments of, on average, 700 bp as assessed by bioanalyzer traces (data not shown). Given that most of the novel 5′ exons identified in our previous study were less than 300 bp in total length (Manak *et al.* 2006), we reasoned that capture of 700 bp fragments by known exonic probes would allow for capture of entire 5′ unannotated exons. After elution from the array, the postcapture cDNA was then sequenced using the Roche 454 Genome Sequencer FLX system. The average read lengths obtained were 356 bp for the ovary sample and 374 bp for the testes sample, although we were able to obtain 53,791 and 48,218 reads over 500 bp for each sample, respectively (with the longest reads of 703 and 686 bp).

### Identification of novel exons and splicing events

**Figure 2:**
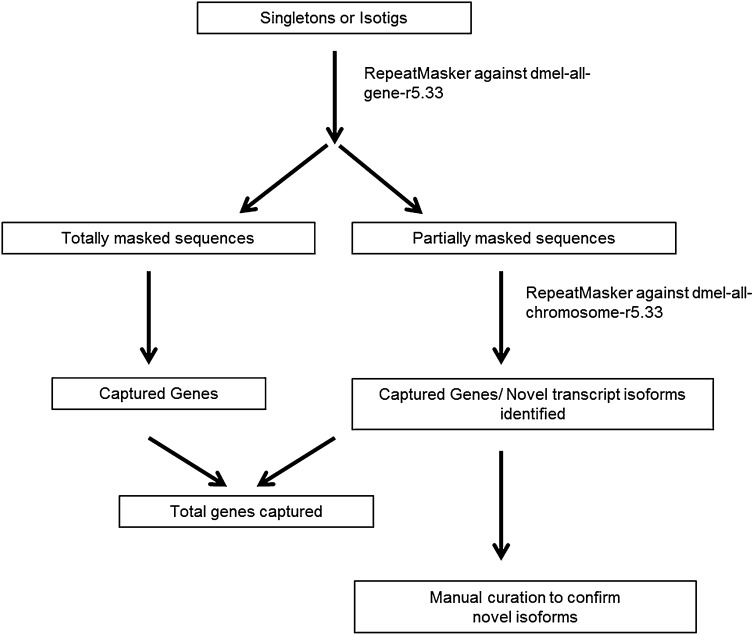
Identification of novel transcript isoforms. RepeatMasker was used to screen singletons and isotigs against *D. melanogaster* all-gene database (dmel-all-gene-r5.33.fasta.gz). Partially masked sequences were then repeat masked against dmel-all-chromosome-r5.33. Total genes captured are a combination of these two categories. Novel transcript isoforms were confirmed by hand curation.

Over 540,000 and 700,000 reads were generated for the ovaries and testes cDNA samples, respectively. Isotigs and singletons were generated using Newbler (v2.3) and then mapped back to the genome (Table 1). This resulted in the creation of 9553 and 13,275 isotigs (sequences built from multiple reads that are analogous to individual transcripts) as well as 16,554 and 20,037 singletons (single read sequences) for the ovaries and testes, respectively (Table 1). The RepeatMasker was used to determine whether the expressed transcripts contained novel, unannotated transcript information. Through repeat masking, novel transcribed units appended to known exons represented on the array were identified and subsequently hand-curated for validation (Figure 2). To call a transcribed unit as a novel exon, we required perfect splice consensus sites in the genomic sequence flanking the exon.

**Table 1:** CoNECT Summary Statistics

	Ovaries	Testes
Target bases covered	14,137,838	17,226,214
Percent target bases covered	37.8	46.1
Target bases not covered	23,222,025	20,133,649
Percent target bases not covered	62.2	53.9
Total number of reads	549,729	703,613
Number of reads in target regions	543,373	696,274
Percent of reads in target regions	98.8	99
Number of isotigs assembled by Newbler	9,553	13,275
Number of singletons	16,554	20,037

**Figure 3:**
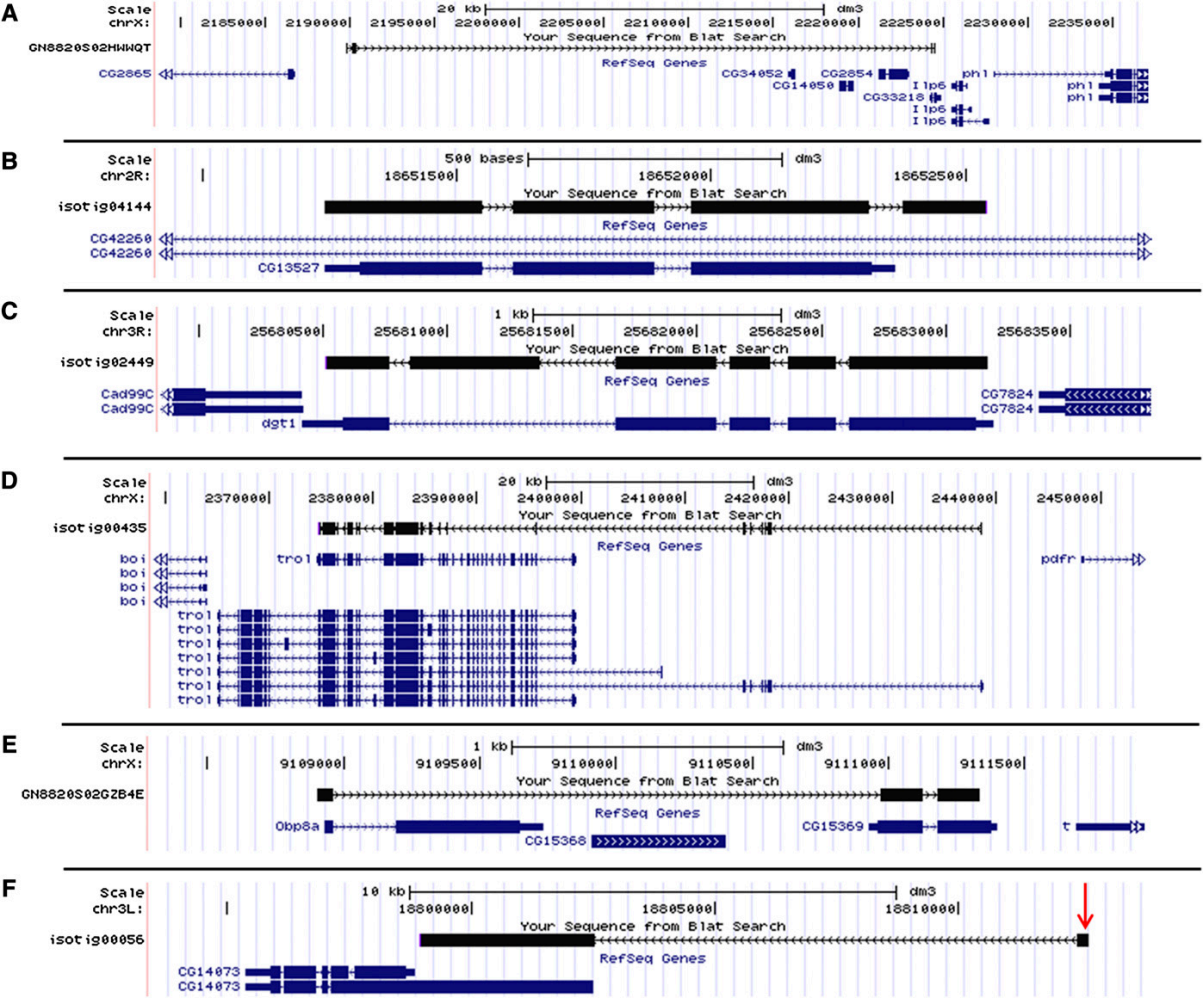
Examples of novel transcript isoforms identified by sequence capture. (A) Testis singleton GN8820S02HWWQT matches to gene CG33218 and contains two 5′ novel exons, which are about 35 kb away from the first annotated 5′ exon. (B) Testis isotig04144 matches to gene CG13527 and contains a 3′ novel exon. (C) Testis isotig02449 matches to gene *dgt1* and contains a novel internal exon. (D) Testis isotig00435 matches to gene *trol* and is missing several internal exons. (E) Testis singleton GN8820S02GZB4E fuses together exons of genes *Obp8a* and CG15369. (F) Ovary isotig00056 matches to gene CG14073 and contains a novel 5′ exon. P-element Dmel\P{GSV6}GS14564 is inserted in the middle of this novel exon.

Seven categories of novel exons or extensions (continuations of the sequence of an annotated exon) were identified, which included 5′ novel exons, 5′ exon extensions, 3′ novel exons, 3′ exon extensions, novel internal exons, internal exon extensions, and gene fusions. Overall, 298 transcripts with novel 5′ exons were identified, thus signifying novel transcription start sites (TSS), some of which are potentially germline specific, with 22 sequences represented by more than one novel exon (Figure 3A and Table S1). The average size of novel 5′ exons was 129 bp and 135 bp for ovaries and testes, respectively, strongly suggesting that our nebulization strategy (which generated cDNA fragments of, on average, 700 bp) would allow capture of most novel first exons. For example, if the associated second (array-interrogated) exon was 100 bp and this exon was bound by a 700 bp cDNA fragment, then up to 600 bp of “extra” sequence could theoretically be “captured.” Sequencing from the novel exon end with the 454 technology would then generate enough sequence to cover the novel first exon and extend into the annotated exon. For example, testes isotig04218 contains a novel exon of 368 bp as well as an extension of the first annotated exon; thus, 484 bp of novel sequence was captured via the exome array. In summary, sequencing with a long-read platform allowed us to faithfully determine precise exon-intron-exon structures over several hundred contiguous nucleotides.

Similarly, we identified 2084 5′ gene extensions that extend the annotated gene in the 5′ direction without interruption by an intron (Table S2); 58 3′ novel exons (Figure 3B and Table S3); 891 3′ gene extensions (Table S4); 279 novel internal exons (Figure 3, C and D, and Table S5), and 1010 internal gene extensions (Table S6). We also identified 10 gene fusions, each of which connects genes that were previously annotated separately (Figure 3E and Table S7). Finally, we mapped 27 P-elements (the lines of which are available at the Bloomington, Kyoto, or Harvard Medical School stock centers) to novel 5′ exons or distal 5′ extensions, thus mapping previously unmatched mutations to their respective genes (Figure 3F and Table S8).

### Capture of annotated germline-specific transcript isoforms

Alternative splicing contributes to sexual differentiation in flies (Baker 1989; Gan *et al.* 2010), and several studies have identified germline-specific isoforms (Gan *et al.* 2010; Graveley *et al.* 2011). Thus, we wanted to verify that our methodology was able to identify known germline-specific isoforms. One such example is represented by the *Reps* gene, which contains one highly conserved exon included in males but skipped in females, consistent with previously characterized isoforms (Figure S1) (Graveley *et al.* 2011). Along the same lines, several genes were identified that expressed novel isoforms in both ovaries and testes, either represented by the same novel isoform or two different gender-specific isoforms (Table S9). For example, in the category of 5′ novel exons, six genes shared the same novel exon in both ovaries and testes (Figure S2A), suggesting that transcription of these isoforms is controlled by common and potentially germline-specific promoters. Two genes expressed different novel 5′ exons in ovaries and testes, suggesting the use of gender-specific transcription start sites. In other cases, we observed germ cell–specific isoforms of the same gene, each gender-specific isoform utilizing a different assortment of either novel or annotated internal exons (for example, see Figure S2B).

### Comparison with tiling array analysis

We compared the ovary capture array data with data generated from tiled genomic microarrays to determine whether our capture methodology could identify a similar expressed gene set. A tiling genomic microarray set designed by us (Nien *et al.* 2011) (50-mer probes tiled approximately every 33 bp) was hybridized with labeled cDNA generated from the polyA-purified mRNA (the same material used for the array capture protocol) and processed to generate gene expression calls using an algorithm previously developed by us (Nien *et al.* 2011) (see *Materials and Methods*). Using this strategy, we have recently shown that tiling arrays are more sensitive in identifying expressed genes than standard gene expression arrays (Nien *et al.* 2011). In addition to algorithmically calculating more statistically robust gene expression values due to increased probe numbers and devaluation of poorly performing probes, we can visualize tiling array data in a genome browser for confirmation of expression. This is particularly useful for assessing transcription of genes in which the interrogated transcript isoform differs from the annotated isoforms either due to partial misannotation of the gene or expression of a novel isoform that omits annotated exons. We previously found that calling a gene as expressed if 70% of the probes were above background was the most reliable measure for determining whether a gene was on or off (Manak *et al.* 2006) (see *Materials and Methods* and Table S10), and we have used these parameters for this dataset. Collectively, 7922 genes were called as expressed for the capture array dataset, and 7981 genes were called as expressed for the tiling array dataset (Table S11). Among the genes called for the capture array dataset, 87% were also called for the tiling array dataset. Conversely, among the genes called for the tiling array dataset, 87.7% were also called for the capture array dataset (Table S11). Therefore, these results show a strong concordance between the datasets and indicate that the sequence capture methodology is capable of capturing a large percentage of the same genes as a tiling array, thereby demonstrating that the capture array probes are effective in binding their targets. It should be noted that deeper sequencing of our captured sample would likely have resulted in more genes being identified by CoNECT; we point out that the depth of sequencing necessary to identify *all* relevant expressed transcripts in a tissue is currently unclear.

We next wanted to determine whether the capture array dataset showed evidence of “normalization” on a genome-wide scale such that lower expressed transcripts were more effectively called at the expense of transcripts that may have saturated the binding of exonic capture probes (see *Discussion*). In particular, we compared FPKM values from our CoNECT study to expression values generated from the tiling array data. As shown in Figure S3A, there is a clear and substantial compression in the range of transcript abundance in our CoNECT data relative to the tiling array data (which spans a far greater overall range). Lower expressed genes are represented by proportionally more reads than higher expressed genes, thereby allowing for more effective interrogation and/or discovery of lower expressed genes while avoiding repeated resequencing of highly expressed genes.

### Enrichment of capture-targeted transcripts

We wanted to determine whether our strategy could effectively enrich for targeted transcripts at the expense of transcripts that were not represented in the capture array design. We thus identified a duplicated gene called *ada1* (*ada1-1*, FBgn0051865; *ada1-2*, FBgn0051866) that showed strong expression on the tiling array but was not interrogated by any probes on the capture array. As the genes are 99% identical at the mRNA sequence level and both are included on the tiling array, the tiling array–based gene expression levels for each can be added together to generate an overall expression level for this gene duplication (as hybridization of labeled cDNA to one gene or the other would be stochastic). To demonstrate that reads from this gene were preferentially excluded from targeted genes expressed at similar levels, we looked at the number of reads generated by 454 sequencing for both *ada1-1*/*ada1-2* and the 10 most closely expressed genes determined via the tiling array analysis, as well as their FPKM values (Table S12; see *Materials and Methods*). In total, 822 reads corresponded to these 10 genes (with an average FPKM value of 47.84/gene), whereas no reads corresponded to *ada1* (with an FPKM value of 0). The absence of reads for *ada1* is strongly significant based on expectations of random distribution among all 11 transcripts (binomial test; *P* < 1 × 10^−9^) once expectations are normalized by the relative length of all the 11 transcripts. Similar results were obtained when we used the median number of reads for the 10 transcripts interrogated by the capture array (*P* < 1 × 10^−9^), which takes into account possible introduced variance in the capturing efficacy. In conclusion, CoNECT can efficiently enrich for specific transcripts at the expense of undesired ones, thus providing more sequencing power focused specifically on transcripts of interest.

### Capture-specific transcriptome

We next compared the ovary capture array data with the tiled microarray expression data to determine whether we could identify mRNA transcripts that were undetectable via microarrays due to either (1) low-level expression in the ovary or (2) expression in a small minority of the cells present in ovary tissue (which would thus be strongly underrepresented in the overall transcript pool), but which were detectable via sequence capture due to selective enrichment of those isoforms. Nine hundred seventy-four genes were called as expressed via array capture but not called as expressed using the tiling array and 70% positive probe threshold (see Figure S4 for two examples). For genes represented on the tiling array that did not have a single probe passing our background threshold after cDNA hybridization and processing (the most severe restriction we could impose), we were still able to identify 18 separate gene transcript sequences that were present in the array capture data and represented by at least two exons (Table S11). Although we identified many more sequenced regions overlapping exons in this dataset, we only considered multi-exon transcripts to be evidence of real transcription, as single exon reads could in theory be produced by genomic DNA contamination. Additionally, it should be noted that after analysis of hybridized arrays, often a subset of the tiling array probes interrogating a gene pass the threshold for being called as expressed, even if the gene is not actually expressed; this is due to the recognized limitation of array technology in which probes can experience spurious cross-hybridization for a subset of the interrogating probes. Thus, the 70% threshold for tiling array gene data are a very important parameter. Nonetheless, these data demonstrate that our capture methodology can identify transcripts below the sensitivity of the tiling array.

One thousand thirty-three genes were called as expressed on the tiling array but were not represented by any sequencing reads from the array capture. These data suggest that the sequencing was not deep enough to capture all the relevant expressed transcripts in the ovary. Indeed, out of 25.9 µg of postcapture amplified cDNA, only 500 ng was used for sequencing (approximately 1/50 of the material), covering a total of nine lanes of the 454 sequencer (9/16 PTP). It should be emphasized that deeper sequencing of our captured sample would not only reveal transcripts called by the tiling array yet missed by the sequencing depth we achieved with this study but also would likely identify additional lower-abundance transcripts not called by the tiling array, in addition to filling out any “holes” in the transcripts that were identified (Figure S4).

We next wanted to determine whether any of the transcripts enriched on the capture array but not represented in the tiling array gene expression list were potentially involved in germ cell development. Indeed, *hu-li tai shao* (*hts*, FlyBase ID: FBgn0263391), a gene encoding an integral membrane protein required for ring canal formation during oogenesis, was identified exclusively by the capture array. Female flies harboring mutations in this gene produce egg chambers that contain less than 15 nurse cells and lack an oocyte (Yue and Spradling 1992). Another capture array–specific transcript is represented by the *benign gonial cell neoplasm* gene (*bgcn*, FlyBase ID: FBgn0004581), which has been shown to be involved in germline stem cell (GSC) maintenance (Li *et al.* 2009b). Mutations in *bgcn* lead to an overproliferation of gonial cells at the expense of gametocyte differentiation (http://flybase.org/reports/FBgn0004581.html). These mutations produce a distinctive “tumorous” or hyperplastic stem cell phenotype caused by symmetric GSC divisions that produce more GSCs (Lavoie *et al.* 1999). *bgcn* transcripts are abundant in males but very rare in females (Ohlstein *et al.* 2000) (transcripts are detected in 5–8 cells at the tip of the germarium); however, we were still able to detect this gene in ovaries. Finally, both *C(2)M* and *corona* (FlyBase IDs FBgn0028525 and FBgn0038612, respectively), which encode synaptonemal complex components necessary for meiotic recombination, were also capture array specific. In wild-type ovaries, *C(2)M* and *corona* are only expressed within a subset of nuclei in regions 2A and 2B of the germarium and within the oocyte nucleus in region 3 and early egg chambers within the vitellarium (Page *et al.* 2008), which are less than 20 cells in each ovariole (Takeo *et al.* 2011). Collectively, these results underscore the power of CoNECT to enrich for transcripts expressed in a minority of cells within a tissue, even when the overall abundance of the transcripts is low and undetectable via gene expression microarray.

The recent study by Gan *et al.* (2010) allows us to directly compare traditional RNA-seq methodologies with CoNECT, because they performed an analysis on poly-A RNA isolated from ovaries as well, using the Illumina/Solexa genome analyzer to perform the RNA-seq. Approximately 30 million reads were generated compared with the approximately 550,000 reads generated in our study. However, even though we generated 55 times fewer reads, 421/974 capture array–specific genes (as determined for our tiling array dataset comparison) were not identified as expressed by the traditional RNA-seq (Table S11).

We also determined which genes called as expressed by CoNECT were also called as expressed by Gan *et al.* (2010) (6700/7922, or 84.57%), as well as which genes called as expressed by Gan *et al.* (2010) were also called as expressed by CoNECT (6700/8434, or 79.44%). These data demonstrate that there is a strong concordance between the datasets and that the sequence capture methodology is capable of capturing a large percentage of the same genes as traditional RNA-seq.

Finally, we compared the genes called as expressed by both CoNECT and Gan *et al.* (2010), and we identified over 1000 genes (1222 in total) that were exclusively called by CoNECT compared with the standard RNA-seq protocol (Table S11). Although we recognize that there may have been some variability in both biological factors (strain differences, age, temperature, food, etc.) and technical factors (ovary dissection, RNA purification, and cDNA synthesis) between the two studies, the data nonetheless demonstrate that CoNECT is able to identify additional transcribed genes that were not identified using a more traditional RNA-seq approach, with much fewer overall sequencing reads.

### Identification of a putative transcriptional signature

To determine which functional gene classes were present or statistically enriched in the capture-specific gene list, we performed functional annotation clustering gene ontology (GO) analysis utilizing the David Bioinformatics Resources (Huang da *et al.* 2009). Several gene classes (42 clusters) were identified that had highly significant enrichment *P*-value scores (less than 0.05; Table S13), as well as a significant number of classes with higher *P*-values but nonetheless informative regarding pathway analysis. For example, only a small number of neurons (two pairs of nerves) innervate the ovary, specifically the peritoneal sheath and lateral oviduct (Middleton *et al.* 2006), and yet CoNECT was able to identify a number of transcripts that carry out important roles in neurons (Table S14). Some fell into statistically significant neuronal classes (12 genes), whereas others fell into nonsignificant classes (51 genes). Additionally, several genes were identified on the capture-specific list that were expressed in neurons but did not fall into any GO classes (13 genes, termed “additional neuronal genes”; Table S14). Although we are uncertain how many of these genes are specific to this cell type, at least one of them (*Vmat*, the *Drosophila* vesicular monoamine transporter) is expressed in all of the ovary-innervating neurons and in none of the other cell types in the ovary (Middleton *et al.* 2006; Christopher Elliott, personal communication), providing definitive proof that a transcript from this specific cell-type could be identified by CoNECT. Other identified genes include those that play primary, if not exclusive, roles in neuronal cells (*e.g.*, *SoxNeuro*, *late bloomer*, and *highwire*). For example, antibody staining of the synaptic growth-controlling protein *highwire* in embryos and larvae showed exclusive expression in neurons, resolving into expression in the presynaptic terminals, including those at neuromuscular junctions (NMJ) (Wan *et al.* 2000). We note that the neurons that innervate the ovary form NMJs with ovary musculature. Moreover, *late bloomer* (which encodes a neural tetraspanin involved in synapse formation) is expressed almost exclusively in motor neurons during embryogenesis, with the remaining expression observed in sensory neurons (Kopczynski *et al.* 1996). Finally, *SoxNeuro*, a gene encoding an HMG-box transcription factor, is not only expressed in neurons but also appears to be required for maintenance of neural tissue (Buescher *et al.* 2002).

We reasoned that (out of all the other non-neuronal ovary cell types) the most likely cell type to express these “neuronal signature” genes would be the oocyte, given that maternal RNAs are deposited there by nurse cells to support early embryonic development, including the early stages of neurogenesis. We thus accessed expression data from 0–2 hr embryos (which would consist primarily of maternal message) from our previous study (Manak *et al.* 2006). Significantly, only 2 out of the 76 genes in the “neuronal signature” were called as expressed in these embryos (Table S14).

To address the possibility that some of the genes called by CoNECT might be associated with ovary tissue that was contaminated with other tissues during dissection (an unlikely, but possible, scenario given the relative ease with which ovaries can be dissected from adult females), we looked at whether genes exclusively expressed in a neighboring tissue were called by CoNECT. We chose to look at five genes previously shown to be hindgut-specific [CG9993, FBgn0034553; CG13215, FBgn0033592; CG13129, FBgn0083945; CG12826, FBgn0033207; CG17781, FBgn0039196; (Chintapalli *et al.* 2007)]. None of these genes was represented by reads generated from CoNECT, suggesting that the ovary preparations were pure. Nevertheless, we cannot entirely rule out the possibility that some reads in our dataset came from small amounts of contaminating tissues. Collectively, these data suggest that CoNECT is able to identify a transcriptional signature specific to the neurons that innervate the ovary, although it is possible (and perhaps probable) that some genes within the “neuronal signature” are expressed in other cell types in the ovary.

We directly compared the CoNECT dataset (Table S16) with the RNA-seq dataset by Gan *et al.* (2010) genome-wide and generated a scatter plot of FPKM values produced by the two methodologies (Figure S3B). Similar to what was seen for the tiling array dataset comparison, we observed a “normalization” effect, in which a quadratic fit was better than a linear one (*P* = 0.002). Thus, lower expressed genes are represented by proportionally more reads than higher expressed genes for CoNECT compared with traditional RNA-seq data, with a statistically significant tendency for CoNECT to avoid highly expressed genes.

## Discussion

Using an array-based sequence capture and enrichment strategy followed by long-read sequencing, we have identified large numbers of novel exons, splice sites, and transcription start sites in both testes and ovaries of the fruit fly. We also provide a list of available fly lines containing P-element mutations that target novel first exons we have identified (Table S8), which should be useful to fly researchers studying these genes. This study provides proof of principle for more directed future studies that seek to enrich more specific types of transcripts. For example, the capture and sequencing of transcriptionally complex splice isoforms of a gene, each expressed in small subsets of cells, would be an obvious extension of our study. *Drosophila* *Dscam* can potentially encode over 38,000 isoforms, and these isoforms are expressed in predominantly one tissue type (namely, the nervous system). Without ultra-deep sequencing of nervous system tissue, the true diversity of transcript isoforms could not be elucidated. By using a sequence capture array that only targets *Dscam*, a more comprehensive cataloging of the expressed isoforms can be undertaken. Interestingly, we identified two partial transcripts in testes that aligned to *Dscam* and represent novel isoforms that had not been annotated before, indicating that these are potential testes-specific *Dscam* transcripts (Table S15) that may be associated with the neurons that innervate the musculature of the testes.

We chose the 454 platform for our high-throughput sequencing over other available platforms for several reasons. First, the 454 system has been shown to work well with longer input sequence fragments. Because the cDNA nebulization step produced products with an average length of 700 bp, we were confident that the platform would generate high-quality sequences. Second, and most importantly, we wanted to use a system capable of long reads so that we could easily and unambiguously annotate any novel exon structures, including their connectivities with annotated (or other unannotated) exons. We recognize that the Illumina paired-end technology has been substantially developed over the last couple years, and this type of platform could be used for further studies in conjunction with array capture.

This study demonstrates that the CoNECT methodology can identify genes exclusively expressed in a small subset of the overall cell population of a given tissue. For example, genes expressed in two pairs of nerves in the *Drosophila* ovaries (*e.g.*, *Vmat*) were identified with CoNECT, even though the neurons make up less than 0.01% of the total cell number in the ovary [over 100,000 nonneuronal cells, including stem, follicle, and nurse cells, are present in a developing ovary (Spradling 1993)]. Along the same lines, both *C(2)M* and *corona* (two other genes identified by CoNECT) encode important meiosis-specific synaptonemal complex proteins and are only expressed in about 20 cells per ovariole. It is important to note that these genes and other classes of genes represented by lower overall sequencing read numbers are not necessarily genes expressed at low levels; their transcripts, even if expressed highly in a few cells, may simply be underrepresented in the overall transcript pool due to the largely nonneuronal cell population. Therefore, many of the genes identified by CoNECT that are expressed in smaller cellular populations of a tissue may in fact be important primary regulators of cellular function. By enrichment strategies followed by gene ontology analysis as outlined above, specific gene classes can be identified, even without prior knowledge of which cell types are present in the tissue of interest. Importantly, without enrichment strategies such as CoNECT, transcriptomics of minority cell types would be difficult. This methodology, alongside traditional RNA-seq (Gan *et al.* 2010; Graveley *et al.* 2011) and locus-directed capture strategies, such as those detailed in Mercer *et al.* (2012), provides a means to more faithfully annotate biologically important transcripts expressed in all layers of a tissue of interest across the entire genome. Several exome capture platforms are already commercially available for a variety of organisms, and these can immediately be employed to probe the desired transcriptome of interest.

It should be emphasized that we chose a whole-genome exome array for these initial studies to provide the proof of principle that our methodology works and to maximize our chances of identifying the largest number of novel exons and splicing events of the *Drosophila* germline, a tissue that has been noted to have a large diversity of transcript isoforms. Nonetheless, future studies will target specific gene transcripts, thus allowing for greater sequencing depth of the relevant transcripts while eliminating any unwanted transcripts from the sequencing step. Indeed, we have shown that the exome array can specifically enrich for genes interrogated on the array at the expense of genes not included in the array design (*e.g.*, *ada1*).

Why have we been able to identify so many additional genes expressed in the ovary? Several possibilities exist. First, we have shown that CoNECT can specifically enrich for lower-expressed transcripts at the expense of higher-expressed transcripts, which is most obvious for the CoNECT/tiling array expression comparison (Figure S3A) but is also seen with the CoNECT/traditional RNA-seq comparison (Figure S3B). This is likely in part due to the effect of some highly expressed transcript cDNAs saturating the available probes on the capture array such that no additional transcripts can be captured, thereby “enriching” for lower-expressed transcripts to a greater extent than higher-expressed transcripts. Second, the nature of the hybridization strategy of the capture protocol may facilitate enrichment of lower-level transcripts even in relation to transcripts that do not saturate their target exonic probes. For example, most transcripts of a gene expressed at moderate-to-high levels will efficiently hybridize to their capture probes, but the kinetics will change such that the hybridization becomes less efficient as time goes on because fewer target probes are available for hybridization. However, this is not the case for lower-expressed transcripts in which there is always an abundance of capture probes available for hybridization. Thus, over time, hybridization of lower-level transcripts would be favored over transcripts from more highly expressed genes. Third, given that highly repeated rRNA genes were omitted from the capture array design, we were able to more comprehensively interrogate the transcripts of interest, which by definition, would allow for better interrogation of low-level transcripts. Whatever the case, CoNECT was able to identify a great many additional genes as being expressed compared with either the traditional RNA-seq dataset (with 55-fold fewer reads) or with the tiling array gene expression dataset.

## Supplementary Material

Supporting Information
