# Supplementary material for: Microarray-Based Capture of Novel Expressed Cell Type–Specific Transfrags (CoNECT) to Annotate Tissue-Specific Transcription in Drosophila melanogaster
Source: G3 (Bethesda). 2012 Aug 1;2(8):873–82. doi: 10.1534/g3.112.003194 (PMC3411243; doi:10.1534/g3.112.003194)
Supplement: Supporting Information [file supp_2_8_873__index.html]

Supporting Information 

# Microarray-Based Capture of Novel Expressed Cell Type–Specific Transfrags (CoNECT) to Annotate Tissue-Specific Transcription in *Drosophila melanogaster*

## Supporting Information for Hong *et al.*, 2012

**Files in this Data Supplement:**

- Supporting Information - Figures S1-S4, Files S1-S7, and Tables S1-S16 (PDF, 778 KB)
- Figure S1 - Testis- and ovary- specific isoforms of the *Reps* gene (PDF, 167 KB)
- Figure S2 - Transcripts with potential germ line promoters or differential germ line exon usage (PDF, 321 KB)
- Figure S3 - A. Scatter plot comparing FPKM values of CoNECT transcripts (Y axis) versus expression values generated from the tiling array (X axis). B. Scatter plot comparing FPKM values of CoNECT transcripts (Y axis) versus FPKM ovary data from (Gan et al. 2010) (X axis) (PDF, 145 KB)
- Figure S4 - Array capture can identify transcripts below the sensitivity of the tiling array (PDF, 284 KB)
- File S1 - ovaries-all-singletons.txt.fna (.zip, 1.5 MB)
- File S2 - ovaries-isotigs\_less 200.txt.fna (.zip, 4 KB)
- File S3 - testes-all-singletons.txt.fna (.zip, 2.2 MB)
- File S4 - testes-isotigs\_less 200.txt.fsa (.zip, 3 KB)
- File S5 - 081229\_Dm\_JM\_Annotations.rar (.zip, 5.9 MB)
- File S6 - 081229\_Dm\_JM\_ChIP\_1\_HX1.rar (.zip, 24.9 MB)
- File S7 - 081229\_Dm\_JM\_ChIP\_2\_HX1.rar (.zip, 26.3 MB)
- Table S1 - 5' novel exons of ovaries and testes (.xlsx, 23 KB)
- Table S2 - 5' extensions of ovaries and testes (.xlsx, 62 KB)
- Table S3 - 3' novel exons of ovaries and testes (.xlsx, 12 KB)
- Table S4 - 3' extensions of ovaries and testes (.xlsx, 33 KB)
- Table S5 - Novel internal exons of ovaries and testes (.xlsx, 18 KB)
- Table S6 - Internal exon extensions of ovaries and testes (.xlsx, 36 KB)
- Table S7 - Gene fusions of ovaries and testes (.xlsx, 11 KB)
- Table S8 - P-elements in novel exons of ovaries and testes (.xlsx, 14 KB)
- Table S9 - Genes with novel transcripts in both ovaries and testes (.xlsx, 22 KB)
- Table S10 - Tiling array gene expression of ovary (.xlsx, 1.3 MB)
- Table S11 - Gene list of Ovary Seq-cap\_Tiling array\_Gan *et al*. (.xlsx, 5.5 MB)
- Table S12 - Captured vs non-captured genes (.xlsx, 12 KB)
- Table S13 - Capture-specific gene enrichment (.xlsx, 60 KB)
- Table S14 - Neuronal genes enriched by CoNECT (.xlsx, 17 KB)
- Table S15 - Gene list of Testis Seq-cap (.xlsx, 157 KB)
- Table S16 - CoNECT\_454\_FPKM (.xlsx, 371 KB)
