# Supplementary material for: Microarray-Based Capture of Novel Expressed Cell Type–Specific Transfrags (CoNECT) to Annotate Tissue-Specific Transcription in Drosophila melanogaster
Source: G3 (Bethesda). 2012 Aug 1;2(8):873–82. doi: 10.1534/g3.112.003194 (PMC3411243; doi:10.1534/g3.112.003194)
Supplement: Supporting Information [file supp_2.8.873_003194SI.pdf]

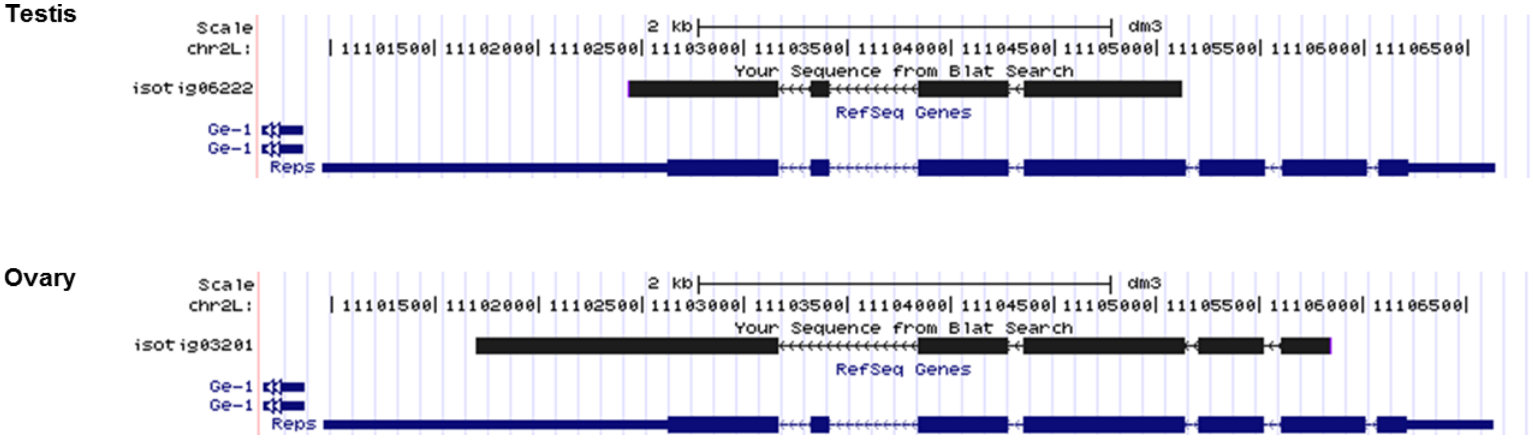

Figure S1 Testis- and ovary- specific isoforms of the *Reps* gene. Testis isotig06222 and ovary isotig03201 match to *Reps*. The sixth exon is a male-specific alternative exon and is skipped in females.

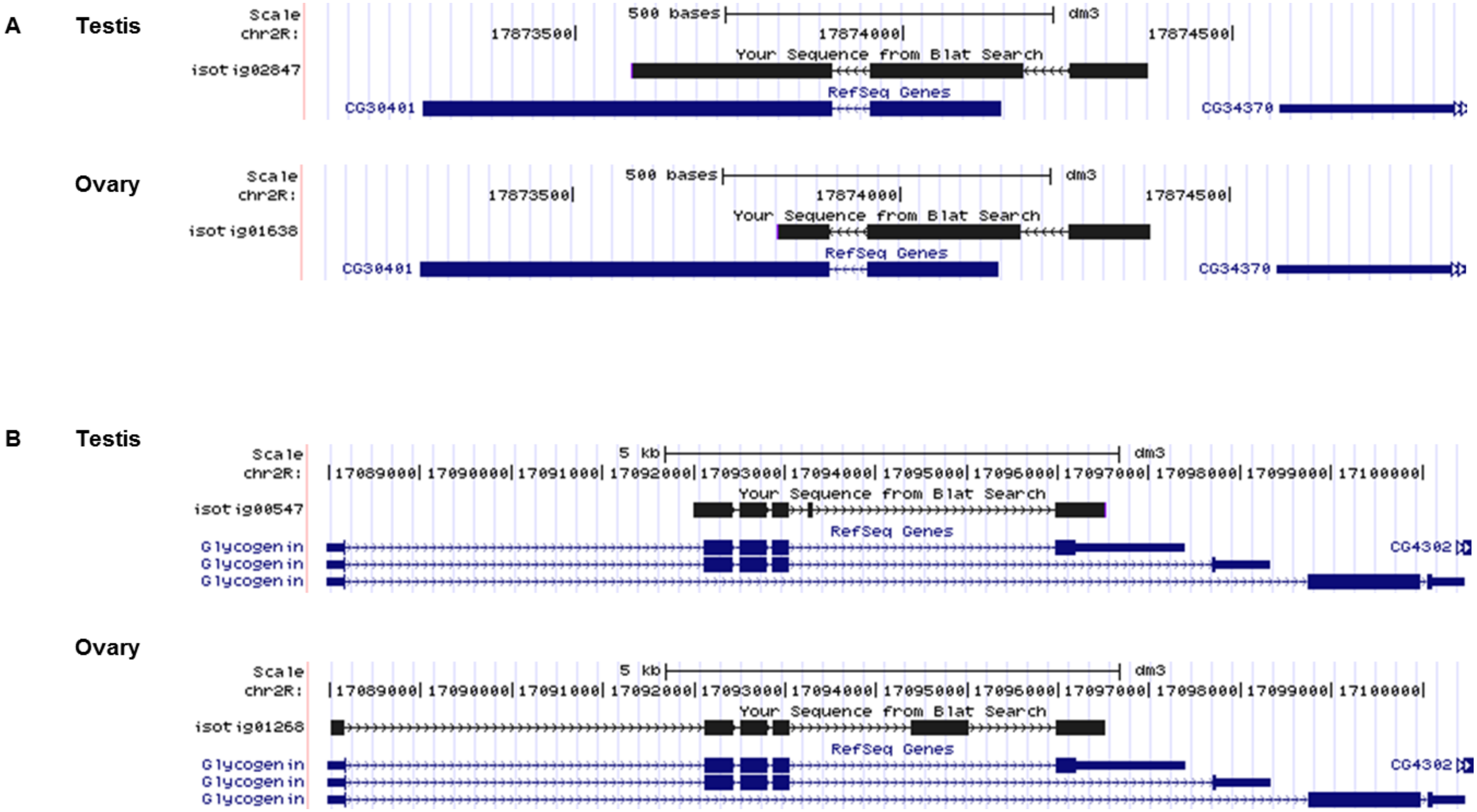

Figure S2 Transcripts with potential germ line promoters or differential germ line exon usage.

(a) Both testis isotig02847 and ovary isotig01638 match to gene CG30401 and have similar novel transcription start sites.

(b) Both testis isotig00547 and ovary isotig01268 match to the gene *Glycogenin* but have different novel internal exons. Additionally, the testis-specific transcript contains an extension of the annotated second exon which may define a novel transcription start site.

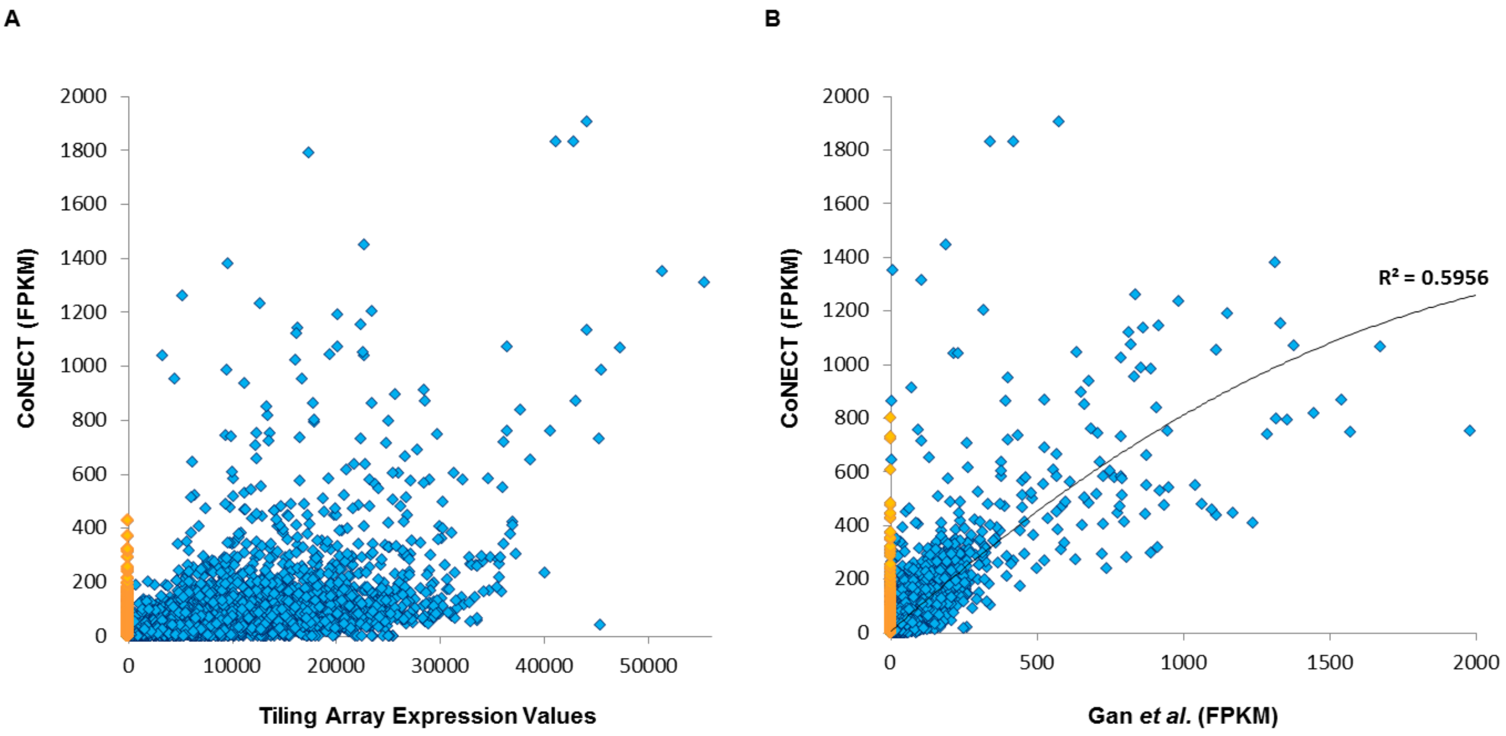

Figure S3 **A.** Scatter plot comparing FPKM values of CoNECT transcripts (Y axis) versus expression values generated from the tiling array (X axis). Note that both the FPKM and tiling array expression values are plotted on a linear scale. Gene transcripts called exclusively by CoNECT are indicated in orange. **B.** Scatter plot comparing FPKM values of CoNECT transcripts (Y axis) versus FPKM ovary data from (Gan *et al.* 2010) (X axis). Gene transcripts called exclusively by CoNECT are indicated in orange.

A

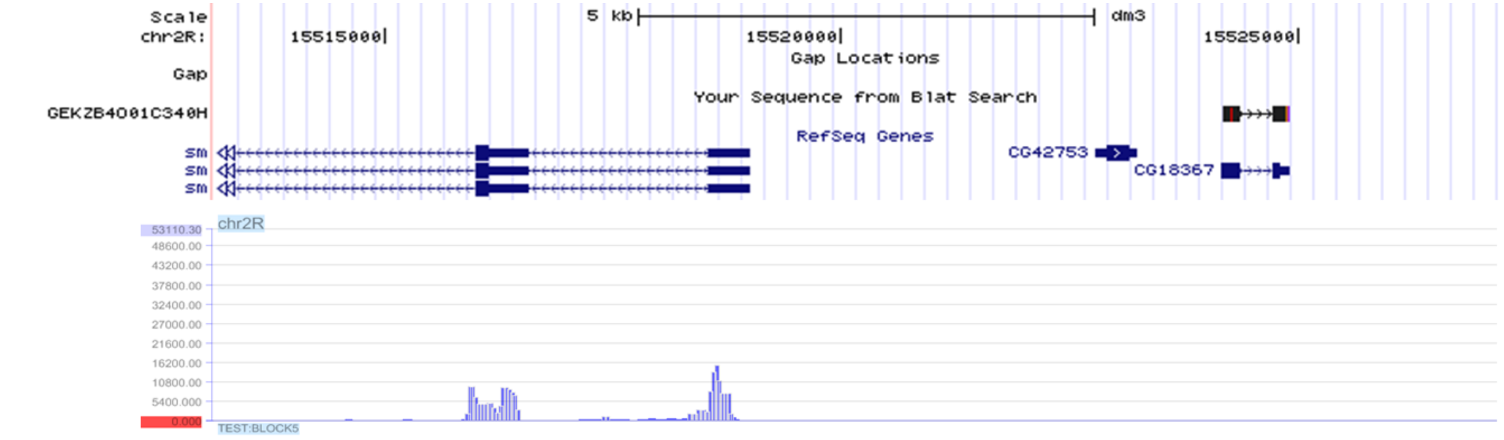

B

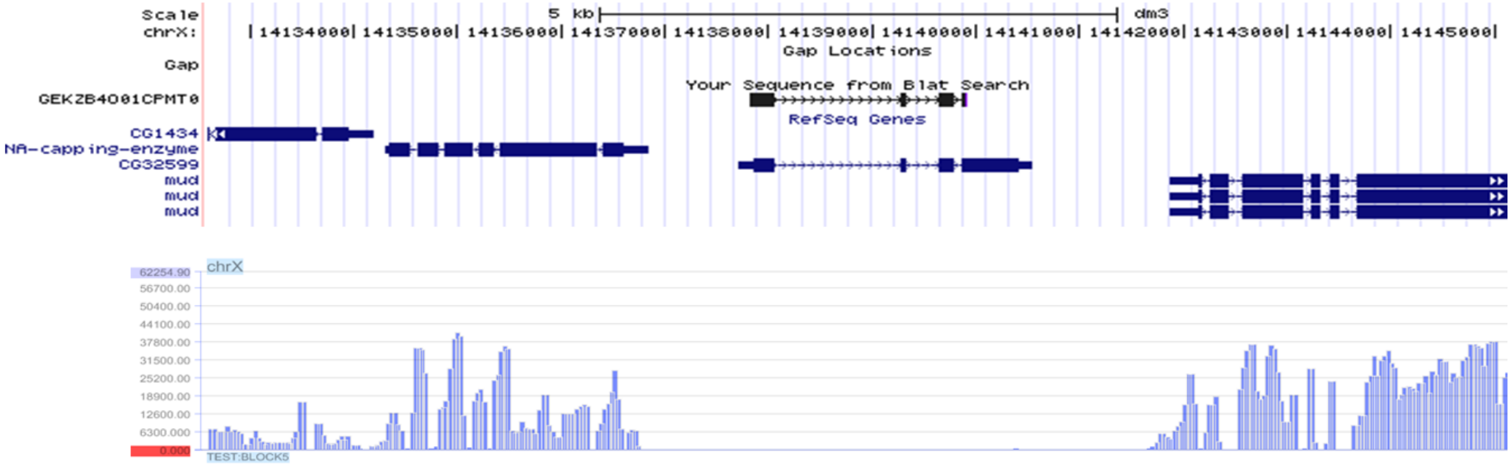

Figure S4 Array capture can identify transcripts below the sensitivity of the tiling array.

(a) Ovary singleton GEKZB4O01C340H matches to gene CG18367 (FBgn0034460) whereas none of the tiling array probes interrogating this gene show positive signals that pass the background threshold.

(b) Ovary singleton GEKZB4O01CPMT0 matches to gene CG32599 (FBgn0260482) whereas only one tiling array probe interrogating this gene shows a positive signal that passes the background threshold.

Files S1-S7

Supporting data

Files S1-S7 are available for download at http://www.g3journal.org/lookup/suppl/doi:10.1534/g3.112.003194/-/DC1.

File S1: ovaries-all-singletons.txt.fna
File S2: ovaries-isotigs_less 200.txt.fna
File S3: testes-all-singletons.txt.fna
File S4: testes-isotigs_less 200.txt.fsa
File S5: 081229_Dm_JM_Annotations.rar
File S6: 081229_Dm_JM_ChIP_1_HX1.rar
File S7: 081229_Dm_JM_ChIP_2_HX1.rar

Tables S1-S16

Supporting tables

Tables S1-S16 are available for download at http://www.g3journal.org/lookup/suppl/doi:10.1534/g3.112.003194/-/DC1.

Table S1: 5' novel exons of ovaries and testes.xlsx

Table S2: 5' extensions of ovaries and testes.xlsx

Table S3: 3' novel exons of ovaries and testes.xlsx

Table S4: 3' extensions of ovaries and testes.xlsx

Table S5: Novel internal exons of ovaries and testes.xlsx

Table S6: Internal exon extensions of ovaries and testes.xlsx

Table S7: Gene fusions of ovaries and testes.xlsx

Table S8: P-elements in novel exons of ovaries and testes.xlsx

Table S9: Genes with novel transcripts in both ovaries and testes.xlsx

Table S10: Tiling array gene expression of ovary.xlsx

Table S11: Gene list of Ovary Seq-cap_Tiling array_Gan et al.xlsx

Table S12: Captured vs non-captured genes.xlsx

Table S13: Capture-specific gene enrichment.xlsx

Table S14: Neuronal genes enriched by CoNECT.xlsx

Table S15: Gene list of Testis Seq-cap.xlsx

Table S16: CoNECT_454_FPKM.xlsx
