# Supplementary material for: Microarray-Based Capture of Novel Expressed Cell Type–Specific Transfrags (CoNECT) to Annotate Tissue-Specific Transcription in Drosophila melanogaster
Source: G3 (Bethesda). 2012 Aug 1;2(8):873–82. doi: 10.1534/g3.112.003194 (PMC3411243; doi:10.1534/g3.112.003194)
Supplement: Supporting Information [file supp_2.8.873_FigureS1.pdf]

Testis

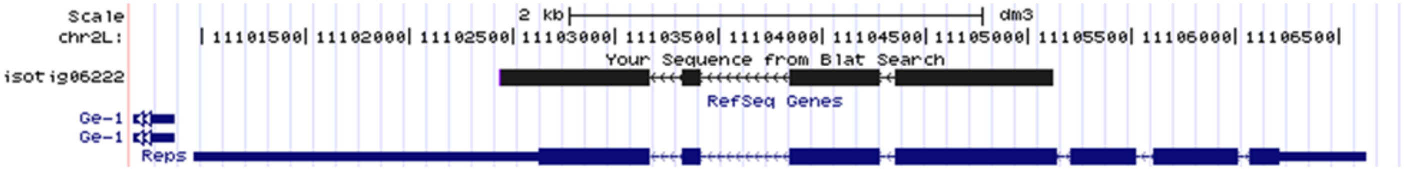

Ovary

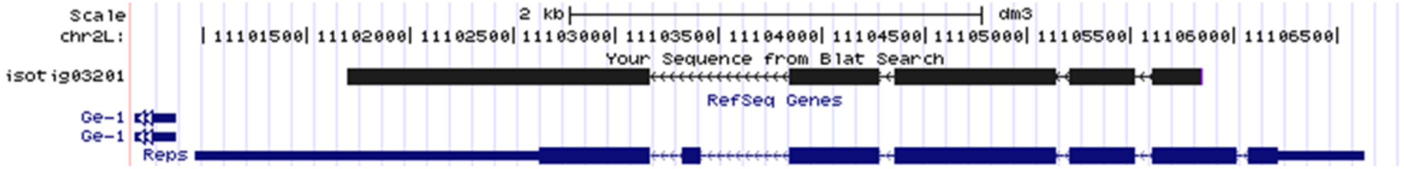

Figure S1 Testis- and ovary- specific isoforms of the *Reps* gene. Testis isotig06222 and ovary isotig03201 match to *Reps*. The sixth exon is a male-specific alternative exon and is skipped in females.
