# Supplementary material for: Microarray-Based Capture of Novel Expressed Cell Type–Specific Transfrags (CoNECT) to Annotate Tissue-Specific Transcription in Drosophila melanogaster
Source: G3 (Bethesda). 2012 Aug 1;2(8):873–82. doi: 10.1534/g3.112.003194 (PMC3411243; doi:10.1534/g3.112.003194)
Supplement: Supporting Information [file supp_2.8.873_FigureS2.pdf]

A Testis

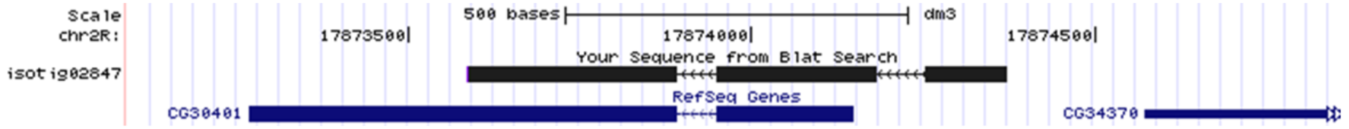

Ovary

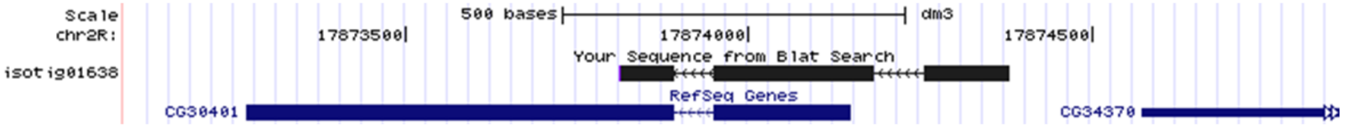

B Testis

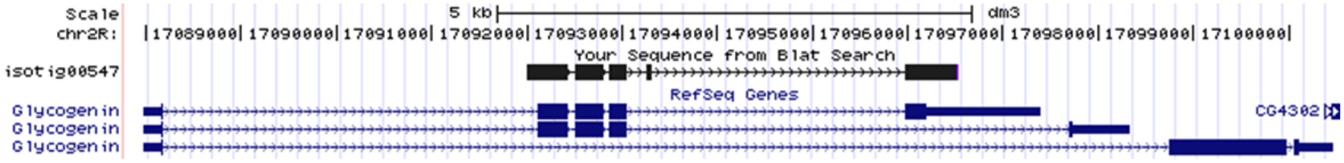

Ovary

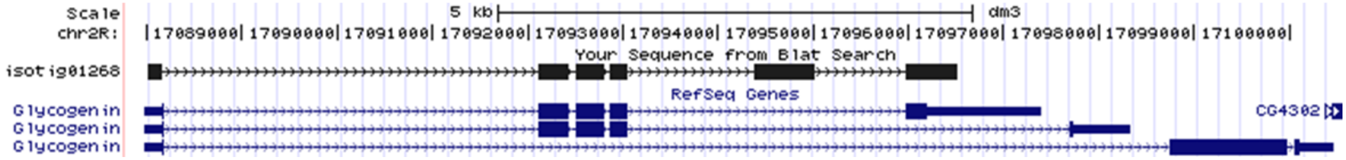

Figure S2 Transcripts with potential germ line promoters or differential germ line exon usage.

(a) Both testis isotig02847 and ovary isotig01638 match to gene CG30401 and have similar novel transcription start sites.

(b) Both testis isotig00547 and ovary isotig01268 match to the gene *Glycogenin* but have different novel internal exons. Additionally, the testis-specific transcript contains an extension of the annotated second exon which may define a novel transcription start site.
