# Supplementary material for: Microarray-Based Capture of Novel Expressed Cell Type–Specific Transfrags (CoNECT) to Annotate Tissue-Specific Transcription in Drosophila melanogaster
Source: G3 (Bethesda). 2012 Aug 1;2(8):873–82. doi: 10.1534/g3.112.003194 (PMC3411243; doi:10.1534/g3.112.003194)
Supplement: Supporting Information [file supp_2.8.873_FigureS3.pdf]

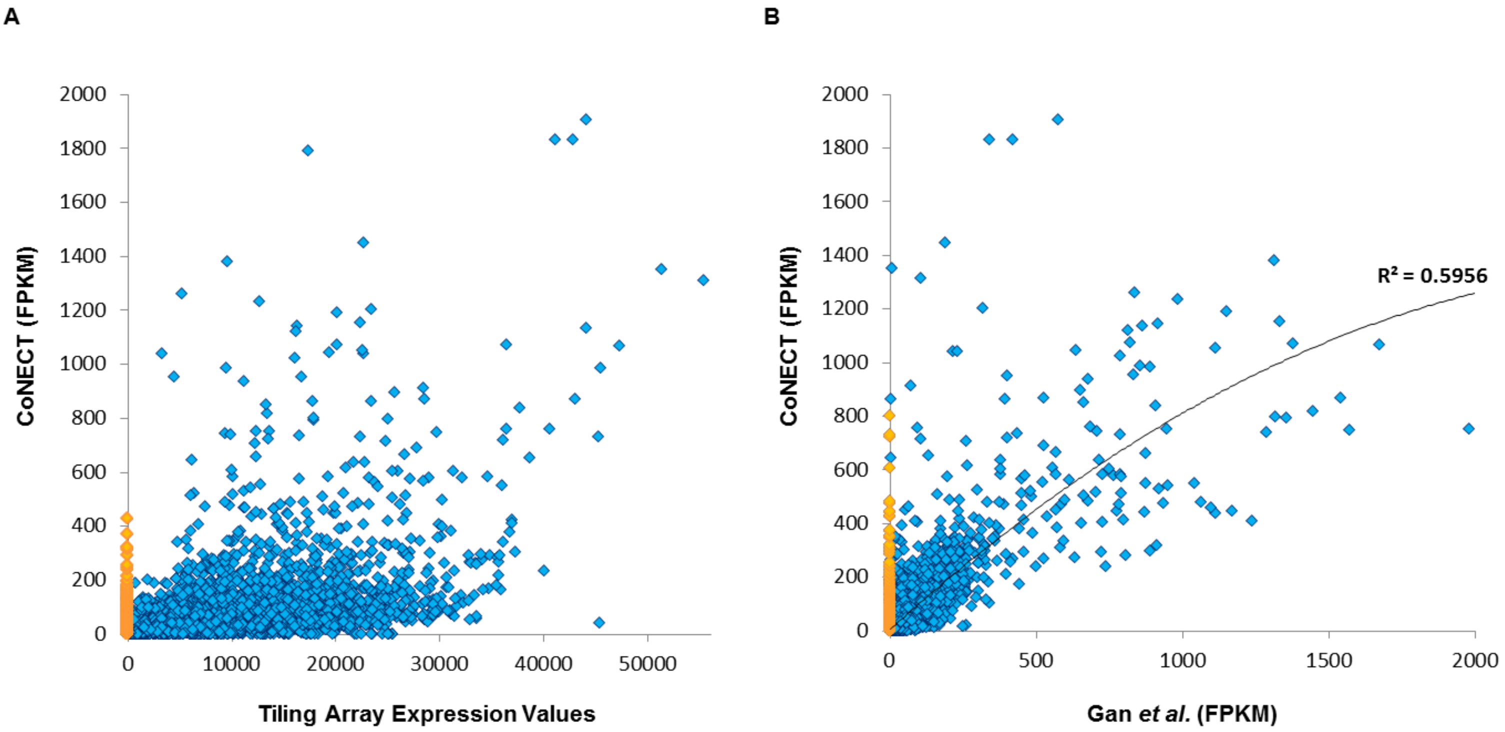

Figure S3 **A.** Scatter plot comparing FPKM values of CoNECT transcripts (Y axis) versus expression values generated from the tiling array (X axis). Note that both the FPKM and tiling array expression values are plotted on a linear scale. Gene transcripts called exclusively by CoNECT are indicated in orange. **B.** Scatter plot comparing FPKM values of CoNECT transcripts (Y axis) versus FPKM ovary data from (Gan *et al.* 2010) (X axis). Gene transcripts called exclusively by CoNECT are indicated in orange.
