# Supplementary material for: Microarray-Based Capture of Novel Expressed Cell Type–Specific Transfrags (CoNECT) to Annotate Tissue-Specific Transcription in Drosophila melanogaster
Source: G3 (Bethesda). 2012 Aug 1;2(8):873–82. doi: 10.1534/g3.112.003194 (PMC3411243; doi:10.1534/g3.112.003194)
Supplement: Supporting Information [file supp_2.8.873_FigureS4.pdf]

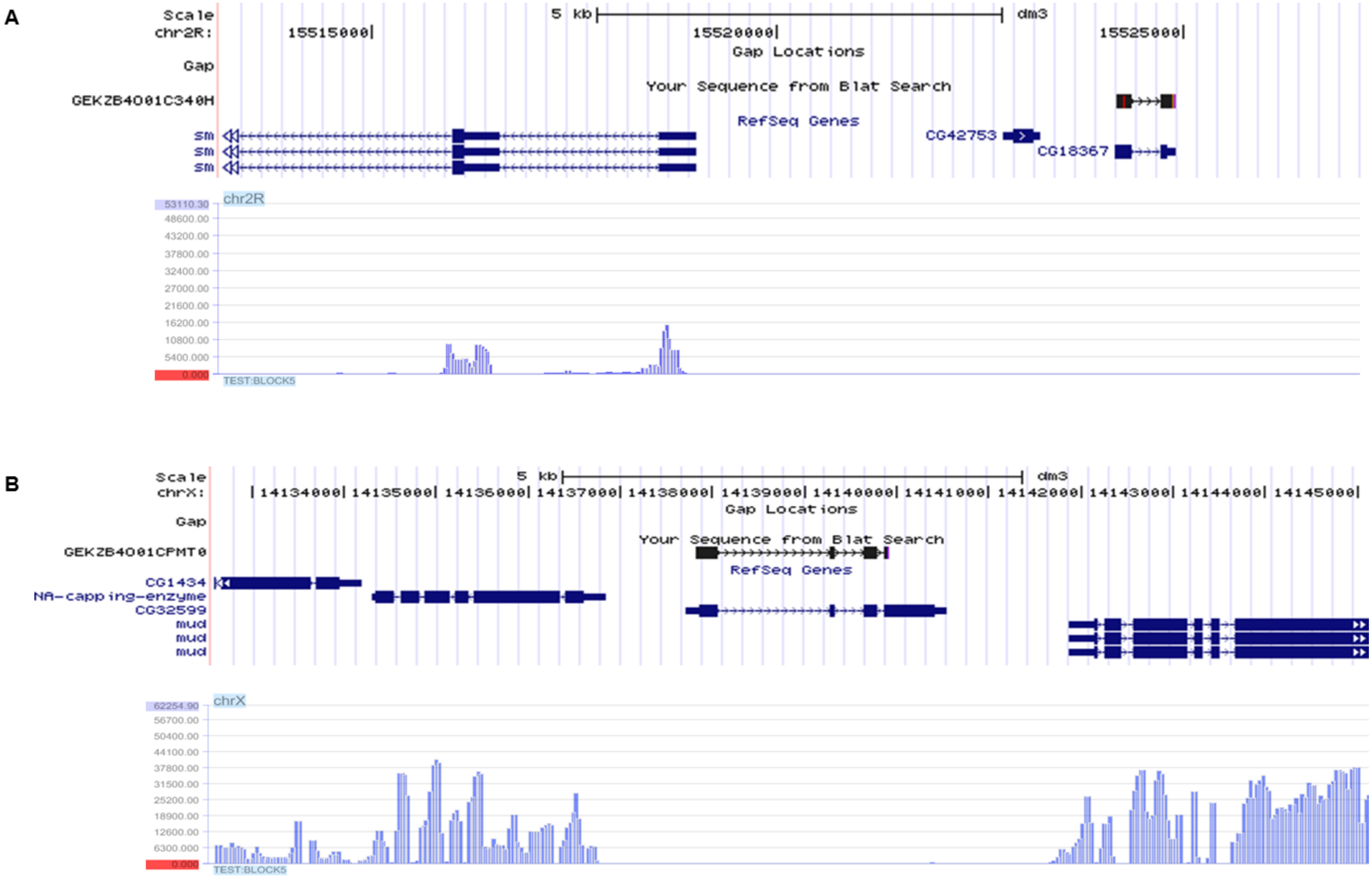

Figure S4 Array capture can identify transcripts below the sensitivity of the tiling array.

(a) Ovary singleton GEKZB4O01C340H matches to gene CG18367 (FBgn0034460) whereas none of the tiling array probes interrogating this gene show positive signals that pass the background threshold.

(b) Ovary singleton GEKZB4O01CPMT0 matches to gene CG32599 (FBgn0260482) whereas only one tiling array probe interrogating this gene shows a positive signal that passes the background threshold.
